# Abscess of the Brain, with Cases

**Published:** 1887-12

**Authors:** W. F. Westmoreland

**Affiliations:** Atlanta, Georgia


### ABSCESS OF THE BRAIN, WITH CASES.

BY W. F. WESTMORELAND, JR., M. D., ATLANTA, GEORGIA.

Wm. C——, colored, age 30, school teacher, in April, 1879, was struck on the head with a common weeding hoe. Applied at once to a physician who dressed the wound; after which the patient was able to walk home. About nine o'clock the same night, became unconscious; the following morning, though better, his mind was greatly impaired; could not talk intelligently. Could neither read, write nor call objects by their names; attempted several times to touch objects, but would miss them. Remained in this condition a month, suffering great pain continually.

Then the pain began to grow worse, and the physician made a cross incision over the wound, and discovered an abscess which discharged a large quantity of blood and pus. About a week after this he found a small hole in the skull, through which the pus was discharged. It was so small there was great difficulty in keeping it open.

In three months from time of accident went to work.

Two years later, in 1881, while in S. C., began to suffer with feelings of pressure on brain and considerable pain. The parts around wound swelled a good deal. Came to Atlanta and a physician opened it. The place discharged for three weeks and healed again.

The peculiar feeling of pressure and pain continued as bad as ever.

But again began work, pain continuing slightly all the time.

During the summer of 1883, again began to suffer from pressure and terrible pains in head and went to Athens, Georgia, for treatment. Then the place was opened and medicine prescribed, but neither did any good. Pain continued to grow worse. Remained in Athens under treatment for three months. Then came to Atlanta and appeared before the surgical clinic of the Atlanta Medical College.

The above history was written for me by the patient after his recovery.

The following history is from clinic record:

Wm. C—— appeared before clinic October 13, 1883. History of syphilis, dating from 1878, and an injury to head dating from 1879. Had been treated for syphilis for several months before injury, and thought himself well. Injury located over upper and posterior part of parietal bone.

*Diagnosis:* Abscess of brain possibly influenced by syphilis, or there may be necrosed bone.

We now cut down on the bone making incision through the old cicatrix. We have reached the bone and find an opening through it that has existed since the injury.

This probe is introduced through the opening and follows a fistulous tract towards the centre of the brain to a depth of four inches. With the probe at this depth the pus flows freely. Discharged about two ounces. Will keep wound in scalp open so as to drain the abscess well. Give him 30 grains of iodide of potassium three times a day on account of syphilitic trouble.

*October 24.*—Patient has suffered greatly, and been in bed once on account of scalp wound closing. Wound re-opened and made larger; kept dilated with elm tent.

*November 28.*—Abscess has been discharging freely; has filled up to three inches. Patient is perfectly free from pain. Treatment continued.

*January 12.*—Wonderfully improved; has gained considerable flesh; feels well; for the first time is free from heaviness in head. Cavity only one and a half inches deep; still a slight discharge.

*February 20.*—Wound entirely healed; suffers no pain. Every symptom has subsided; cavity has filled completely.

Patient discharged as cured, with the admonition to use the specific treatment for sometime.

In March, 1886, patient returned to Atlanta for treatment, with symptoms similar to those he had when he appeared before clinic in 1883. Was put on the same treatment. This treatment was continued until July, 1886, when he came into my hand for treatment. He had made no progress; if anything, was worse. I could introduce a probe three and a half inches downward and forward into his brain. The discharge of pus had decreased to one drachm a day. As there had been no syphilitic symptoms since 1883, I stopped the specific treatment.

I proposed an immediate operation as the only means of relief, which the patient accepted.

On July 9, the discharge of pus suddenly ceased, causing great pain and mental perturbation. Came to my office during the night for relief. I made an incision through the scalp, and could introduce a small probe through the skull, but no pus followed its withdrawal; but it gave him some relief.

*July 11th.*—Pulse 110, temp. 98. Suffered terribly, feeling as if his head would burst; almost crazy with pain, but perfectly rational and able to walk about.

*July 12th.*—Was free from pain; decided to operate next day. Had his head shaved, washed it first with nail brush and soap, then with ether to remove grease, last with a strong solution of bichloride of mercury (1-500), then with a weak solution of the same (1-1000). Then covered his head with bichloride gauze to insure its being antiseptic for the operation.

*July 13th.*—Patient easy; pulse 110, temp. 98½. At 4 o'clock, assisted by Drs. Arbeely, Elkin, Howell and Westmoreland, began operation. Patient was etherized, and his head was thoroughly washed with bichloride sol. (1-1000); strict antiseptic precautions were observed throughout the operation. Instruments were placed in carbolized solution (1-40); towels were wrung out in bichloride sol. (1-2000) and placed around seat of operation; sponges were kept in same solution. During operation the wound was occasionally douched with bichlor. sol. (1-2000).

A crucial incision was made, each incision about two inches long, crossing each other over the fistula. The periosteum was dissected up with the flap. Hemorrhage was very profuse, vessels were ligated with catgut and hemorrhage controlled before trephining was begun. Found the bone healthy, with a slightly grooved fosse ⅛ of an inch wide and nearly ½ an inch long; in the centre of this was a round hole one-tenth of an inch in diameter, passing into the skull in a forward direction. This fosse was situated in the posterior part of the parietal bone on a line with the parietal eminence.

The crown of the trephine was placed so as to include the posterior part of the opening. Trephining was very tedious and progress was slow on account of the irregularity in the thickness of the bone; the anterior part was much thinner and was sawn through much quicker than the posterior; the disc of bone measured ⅜ of an inch on the anterior and ⅝ of an inch on the posterior. Pus began to ooze through before the bone was removed; its removal was followed by a copious flow of pus, followed by blood, five ounces of pus being discharged. The under surface of the disc of bone was necrosed and the underlying dura mater entirely destroyed. The cavity in the brain, from which there was considerable hemorrhage, was very large. I waited a few minutes to see if hemorrhage would cease, but the blood continuing to flow in considerable quantity, I decided to pack it. I washed out the cavity with 1-2000 solution bichlor. and packed it tightly with bichlor. gauze, which effectually stopped the hemorrhage. I did not suture the flaps, but washed the wound with 1-2000 bichlor. sol. powdered with iodoform and dressed with bichlor. gauze and borated cotton.

Patient stood the operation splendidly and came out from the ether perfectly rational and in good spirits.

At 7½ p. m. pulse 80.

At 11½ p. m. pulse 112, temperature 101.

*July 14th.*—8 a. m. Pulse 75, temperature 98½, respiration 18.

" 4:40 p. m. Pulse 98, temperature 98½, respiration 21.

This increase was caused by patient's getting up, contrary to orders, to eat breakfast and dinner.

*July 15th.*—1½ p. m. pulse 98, temperature 99.

Dressed wound; found dressing and packing saturated, and about two ounces of pus and blood flowed from the cavity when I removed the packing; washed out the cavity with 1-2000 bi-chlor. sol.; packed cavity tightly with bichlor. gauze as before to prevent hemorrhage and to insure filling from the bottom. In this case I preferred this way to suturing the flaps and introducing drainage-tubes; powdered the wound with iodoform and dressed as before.

*July 17th.*—Pulse 80, temperature 98½. Dressed to-day same as previously. Temperature and pulse remained normal from this on.

Dressed the wound every three or four days after this until it filled up. At first it filled up very rapidly, the brain expanding and regaining its normal position from which it had been pushed. After this it was slower in filling. Patient's mind clear; was able to think, read or write without any inconvenience from day of operation.

With our present knowledge of the symptoms of cerebral abscess, I think it is impossible to diagnose them, except in very rare cases, or when they are the result of some traumatism that attracts our attention to this as probable.

There is one symptom, however, and I think a very important one, that is present with great regularity, that of *normal or sub-normal temperature;* whereas in other brain troubles, that are apt to be confused with cerebral abscess, the temperature is generally elevated.

The case I operated on the temperature varied from 97½ to 98½, never going over the latter, even when his pulse was at 115 to 120.

Symptoms are too varied to try and describe them, as will be seen in the cases I cite.

The duration of the *latent* stage is generally from one to two months, though there are numerous exceptions. In a case reported, the abscess developed and killed the patient in five days; one at twenty-one years reported by Gerhardt and Schott, and another at twenty-six years reported by Haulin.

Latiencies have been observed during which there were no symptoms of inter-cranial affection. I cite the case reported by Dr. Richardson, of New Orleans, as an interesting example of this class.

Patient admitted to hospital with a wound in the frontal region, which was dressed by house surgeon as a simple scalp wound. Dr. Richardson saw him the day after, when he was suffering no pain, nor did he present any symptoms of brain trouble. Four days after, or fifth day after the injury, he visited the hospital and found the man dead. The patient was suddenly seized with intense headache, followed by coma, and had as suddenly died.

Post mortem examination revealed a slight fissure in the skull, behind which, in the anterior lobe of the brain, there was an abscess holding half an once of pus. There were no symptoms of brain abscess within half an hour of death. This abscess formed without any symptoms and killed the man in five days.

V. W., aged sixty, on September 16th, while fighting, was struck on the head with a hatchet; the blade penetrated the outer table of the frontal and parietal bones about an inch and a half to the left of the sagittal suture, making a clean cut without any depression.

A close examination revealed no fracture of the inner tables.

The man was able to walk two miles to have wound dressed, although there was profuse hemorrhage from the posterior temporal artery. The wound was left open and a carbolized dressing applied. The case progressed favorably; the patient was up in a few days; his temperature and pulse remained normal; he suffered no pain, nor did he have any unpleasant symptoms.

On October 6th he was suddenly seized with a convulsion; on the 13th had another; on the 14th and each succeeding day to the 18th he had two.

On October 18th, Dr. D. H. Howell and myself removed three pieces of bone; as the last piece was removed two or three drachms of pus flowed out; all the rest of the bone was healthy. A probe passed through the opening revealed no cavity of any kind. The operation relieved the feeling of pressure he had had in his head since the first convulsion.

The day after the operation he was free from convulsions and seemed to be doing very well.

On October 20th he was seized with complete hemiplegia of the right side, involving the tongue, and could neither speak nor swallow. He died the next day, October 21st. The temperature of this patient was never *above normal;* autopsy revealed a capsulated abscess the size of a large walnut in the left frontal lobe within the substance of the hemisphere proper, being over a quarter of an inch beneath the surface of the brain. The abscess was connected with a small opening in the dura-mater by a neck, the walls of which were a continuation of the capsule of the abscess below, and were continuous with the dura-mater. The opening in the dura-mater was under the edge of the opening made in the skull by the removal of last piece of bone, which was followed by pus. The fact that the opening was partially under the bone and very small (it would just allow the introduction of a small silver probe) accounts for there being no greater discharge of pus, and was probably the reason our probing did not locate it.

There are several points of interest in this case: First, the short time in which this capsulated abscess formed, it being only twenty days from the time of the accident to time of convulsion.

Second, the convulsions without any premonitory symptoms, and the complete cessation of all head symptoms on the 19th, followed by complete hemiplegia on the 20th and death on the 21st.

Another case, a patient of Dr. R. B. Ridley's, of this city. The injury was the result of a pistol-shot. The bullet entered the frontal bone just above and a little in front of the ear. This man lived thirty-one days. For the first two days he had to have his urine drawn. He had no cerebral symptoms of any kind up to within a few days of his death, was perfectly rational, and able to walk about his room, receive and converse with friends, read, smoke, etc. It getting very warm in the city, he was able to change his quarters, going out some distance to the suburbs. Dr. Ridley tells me that his temperature was never high. I was present at the autopsy; the friends did not want anything done that would disfigure. So no direct examination of the brain was made. An opening was made by removing two or three pieces of bone with a large trephine to search for the bullet. While searching for the bullet an encapsulated abscess was found and removed; it was about the size of a walnut, had very thick walls and a neck about an inch long. I think the neck was longer, as it seems to have been torn, probably, in removing it. The capsule was distended with pus and the bullet was in it.

The *terminal stage* is the beginning of the end and leads to a hasty death.

Meyer's statistics show that cerebral abscesses end in death within a week from the beginning of the *terminal stage.*

But why wait for the terminal stage? It is as much our duty to operate for a cerebral abscess as for one in any other region of the body. More so when we bear in mind the fact that there has *never been a recovery* from a cerebral abscess, the result of traumatism, without operative interference.

Medication does no good, and is simply a waste of valuable time and gives the patient false hopes of recovery.

The operation of trephining, with the strict observance of antisepsis, is by no means a dangerous operation. Briggs has performed the operation thirty times without a death.

Keamer's table shows the following: twenty-five cases of trephining for compound fracture, 2 deaths; 22 cases when the skull was opened through a previously sound scalp for tumors, epilepsy, old depressed fractures, etc., without a death.

When the dura-mater is reached, if the abscess is not in sight cut through it. Frequently, encysted abscess lie beneath; if the abscess is not in sight there are two methods of procedure: First, that of plunging a bistoury into the brain, as was done by Dupuytren and Nélaton with success and Detlmold with partial success, his patient dying later, the abscess opening into the ventricles. It is a bold surgeon that will try this.

Second, the more conservative and better plan, followed by

Hulke, Fenger, Lee and Nancrede in their cases. They explored the brain with an aspirating needle. The first two operators had perfect success in locating and evacuating the abscesses and curing their patients. The aspirating needle should be mounted upon a small syringe, in which withdraw the piston enough to make a vacuum, so that the pus will be drawn into it when the abscess is reached. When started, the needle should be pushed straight in the direction intended, all lateral movement being carefully avoided. In this way a systematic exploration can be made by introducing the needle in different places. When the abscess is located, make an incision into it with a knife, or if you are afraid of hemorrhage, by introducing a pair of stout forceps alongside of the needle, forcibly open them and then withdraw; make a large enough opening for free drainage. Wash out the cavity thoroughly with a 1-2000 bichlor. sol. There is nothing the success of the operation depends on so much as thoroughly efficient drainage. If necessary make a counter-opening. Introduce a couple of fenestrated drainage-tubes, or in some exceptional cases to pack the cavity with bichlor. gauze will be more efficient. When it is possible, close the trephine opening with the disc of bone removed, an opening sufficiently large for the drainage-tube being left. If you expect to replace the bone keep it wrapped in warm carbolized cloths. When strict antisepsis has been carried out, the bone not only retains its vitality, but is an important factor in the process of repair. It has been successfully replaced by Wier and MacEwen. As a coincidence it is noted that in an analysis of 65 cases of cerebral hernia collected by Dr. R F. Weir, post mortems were performed in 27—in 11 of these an abscess was found in the hemisphere corresponding to the injured side, and generally situated immediately beneath the hernia. Dr. Gurdon Buck reports 33 cases, and in every one in which an autopsy was made the same condition of affairs was present.

